# Mediators of Placebo Response to Cannabinoid Treatment in Children with Autism Spectrum Disorder

**DOI:** 10.3390/jcm12093098

**Published:** 2023-04-24

**Authors:** Adi Aran, Moria Harel, Aminadav Ovadia, Shulamit Shalgy, Dalit Cayam-Rand

**Affiliations:** 1 Neuropediatric Unit, Shaare Zedek Medical Center, Jerusalem 9103102, Israel; moriaharel@gmail.com (M.H.); dalitc@szmc.org.il (D.C.-R.); 2 Faculty of Medicine, Hebrew University of Jerusalem, Jerusalem 9112102, Israel; 3 Psychology Department, Yezreel Valley College, Yezreel Valley 1930600, Israel; baminadav@gmail.com (A.O.); shulyshalgy@gmail.com (S.S.)

**Keywords:** autism spectrum disorder, cannabinoids, placebo response, placebo by proxy response, child psychiatry, developmental disorders

## Abstract

The placebo response has a substantial impact on treatment outcome. However, data regarding mediators of the placebo response in children with autism spectrum disorder (ASD) are sparse. This retrospective study investigated possible mediators of the placebo response among participants of a placebo-controlled trial of cannabinoid treatment for behavioral problems in children with ASD (CBA trial, age 5–21 years). We used a specifically designed questionnaire to explore possible mediators of the placebo response in 88 participants of the CBA trial who received a placebo and had valid outcome scores. The parents of 67 participants completed the questionnaire. The placebo response was positively associated with the child’s comprehension of the treatment purpose (*p* = 0.037). There was also a trend for participants who had a relative aggravation of symptoms before treatment onset to improve following placebo treatment (*p* = 0.053). No other domains, including parental expectations, previous positive experience with similar treatments (behavioral conditioning), parental locus of control, quality of the patient–physician relationships, and adherence to study medications were associated with placebo-response. This finding suggests that efforts to explain the treatment purpose to children with disabilities may enhance treatment efficacy in clinical practice and decrease differences in the placebo response between study arms. Contrary to our hypothesis, parental expectations regarding cannabinoid treatment were not associated with the placebo response.

## 1. Introduction

Autism spectrum disorder (ASD) defines a group of heterogenous neurodevelopmental conditions characterized by persistent deficits in social communication and interaction, repetitive and restricted behaviors, and sensory anomalies [1] In the last few decades, numerous pharmacological treatments for these core symptoms of ASD have been investigated, with the majority failing to demonstrate higher efficacy when compared to placebo, in part due to a relatively high placebo response [2,3,4,5,6].

The placebo effect consists of the psychobiological and psychosocial processes that lead to therapeutic benefits in response to administration of inactive treatment. The most established factors underlying the placebo effect are treatment expectation, behavioral conditioning, and the quality of the patient–physician relationship [7,8]. The placebo response comprises all the positive outcomes that follow the administration of a treatment, including the psychobiological and psychosocial processes that underly the placebo effect, as well as spontaneous remission of the disorder regardless of treatment, regression of symptoms to the mean (e.g., in patients seeking help when the disorder peaks), and the effect of being observed (Hawthorne effect; [9]).

The placebo response is an important component of the efficacy of any behavioral or pharmacological treatment. In many commonly used medications, e.g., for pain, depression, and sleep disorders, the placebo response is thought to be responsible for the primary treatment effect [10,11].

Individual differences in the placebo response explain some of the variability in the response of patients to treatments [9]. In placebo-controlled clinical trials, a higher placebo response reduces the relative contribution of the active treatment compared with the placebo, resulting in a key barrier to treatment development due to late-stage failure of initially promising treatments [12,13,14]. At the same time, a higher placebo response can be harnessed to increase treatment efficacy in clinical practice [8]. Hence, identifying mediators of the placebo response in various populations is important to improve the design of clinical trials and enhance treatment efficacy in clinical practice [8,9].

Several studies have demonstrated a relatively high placebo response in pediatric populations, particularly in CNS disorders and trials which use subjective primary endpoints, as is often the case in ASD studies [15]. Indeed, a systemic review and meta-regression analysis of the placebo response in pharmacological and dietary supplement trials addressing the core symptoms of ASD, revealed that 19% of the 2360 participants who received placebo showed either significant or very great significant improvement in their core symptoms following treatment [16]. However, while numerous studies have assessed the mediators of placebo response in adults [17,18,19,20] and typically developing children [21,22], data regarding the mediators of placebo response in children with neurodevelopmental disorders are limited [11].

Furthermore, the treatment outcome in children with disabilities is usually perceived and reported by their proxies and might reflect the feelings of the proxies following the treatment of the participant, regardless of any placebo effect on the participant himself. Subsequently, the placebo response in children with disabilities is often defined as the placebo by proxy response, and its mediators may be different compared with the placebo response in adults and typically developing children.

In a recent paper by Jacob S. et al. [14], the authors discussed the key challenges in ASD treatment development, based on their own experiences of the balovaptan clinical development program, which included three placebo-controlled trials (544 adults and 167 children aged 5–17 years, all with normal IQ). They hypothesized that the placebo response was the main contributor to the observed lack of treatment effect and pointed to three predictors of placebo response in their studies: greater baseline symptom severity, online recruitment of participants, and less experienced or non-academic trial sites. However, specific patient and family characteristics predicting placebo response were not investigated.

In the current study, we aimed to explore child and parent characteristics associated with the placebo response to cannabinoid treatment in a cohort of children and adolescents with ASD and a wide range of functional abilities.

Investigating placebo response is particularly important in studies of cannabinoid treatment. Interest in cannabis and cannabinoid preparations is rapidly growing in both lay and scientific communities, and cannabis preparations are commonly used to alleviate numerous disorders, including chronic pain, chemotherapy-induced nausea and vomiting, depression, and anxiety. Nevertheless, placebo-controlled trials have demonstrated marginal to no efficacy over a placebo for most of the above indications, as seen in recent systematic reviews and meta-analyses (Appendix A; [23,24,25,26,27]).

The higher placebo response in cannabis studies is often attributed to the perceived higher efficacy of medical cannabis compared with most other medications among the general public. However, factors contributing to placebo response in cannabis studies have not been systematically explored so far. We hypothesized that both the child’s comprehension of treatment and the parental treatment expectation would contribute to higher placebo response in children with ASD.

## 2. Materials and Methods

### 2.1. Standard Protocol Approvals and Patient Consent

The CBA trial was a proof-of-concept placebo-controlled trial which assessed the impact of cannabinoid treatment on ASD-associated disruptive behavior, as described previously [28]. The study was conducted between January 2017 and October 2018 in a single referral center for ASD diagnosis and treatment, Shaare Zedek Medical Center, Jerusalem, Israel, and was approved by the local institutional review board and the Israeli Ministry of Health prior to participant enrollment (IRB# 0175-16-SZMC). Participants’ parents provided written informed consent and written consent was also obtained from participants, when appropriate. The protocol of the CBA trial was registered on clinicaltrials.gov as NCT02956226. The current study assessed possible mediators of placebo response in participants of the CBA trial and included secondary analyses of demographic data and outcome measures obtained in the CBA trial, as well as novel data derived from a parent questionnaire that was specifically designed for the current study. The current portion of the study was approved by the Institutional Review Board at Shaare Zedek Medical Center (Jerusalem, Israel; IRB# 0069-22-SZMC) prior to study onset.

### 2.2. The Design of CBA

**Figure 1:**
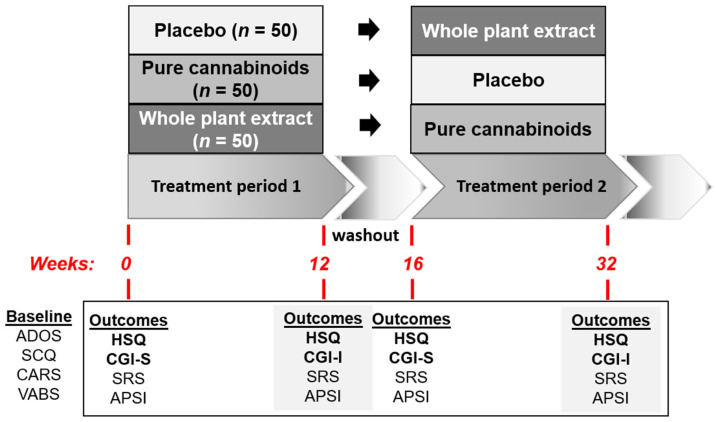
Schematic representation of study design. ADOS—Autism Diagnostic Observation Schedule; APSI—Autism Parenting Stress Index; CARS—Childhood Autism Rating Scale; CGI-S, CGI-I—Clinical Global Impression-Severity, Clinical Global Impression-Improvement; HSQ—Home Situation Questionnaire; SCQ—Social Communication Questionnaire; SRS—Social Responsiveness Scale; VABS—Vineland Adaptive Behavior Scale.

CBA study was a randomized, double-blind, placebo-controlled, cross-over study in 150 children with ASD with 3 treatment options: 2 cannabinoid solutions and one placebo (Figure 1). Participants were randomly assigned (1:1:1 ratio) to receive 1 of 3 treatments for 12 weeks (‘Period-1’). The treatment options were: (1) a whole-plant cannabis extract containing cannabidiol and Δ9-tetrahydrocannabinol in a 20:1 ratio, (2) a purified cannabidiol and Δ9-tetrahydrocannabinol in the same ratio, and (3) a placebo. After a 4-week washout period, participants crossed over to a predetermined second 12-week treatment (‘Period-2’).

The severity of the ASD core symptoms at baseline was determined using the following assessments: Autism Diagnostic Observation Schedule [29]—Second Edition (ADOS-2), a standardized, play-based assessment; the Social Communication Questionnaire (SCQ) Lifetime Form [30], a 40-item, parent-report screening measure; and the Childhood Autism Rating Scale—Second Edition (CARS2; [31]), a quantitative measure of the ASD core symptoms based on a direct behavior observation by a clinician. The adaptive level at baseline was assessed using the Vineland Adaptive Behavior Scales—Second Edition (VABS-2; [32]), a caregiver interview assessing communication, socialization, and daily living skills.

Four outcome measures were used. Two co-primary outcome measures assessed the ASD associated disruptive behaviors: the Home Situations Questionnaire-ASD (HSQ-ASD; [33]), a 24-item parent-rated measure of noncompliant behavior in children with ASD; and the Clinical Global Impression-Improvement scale, with disruptive behavior anchor points (CGI-I; [34]), a clinician ratting scale ranging from 1 (very much improved) through 4 (unchanged), to 7 (very much worse). Two secondary outcomes included the Social Responsiveness Scale—Second Edition (SRS-2; [35]), a 65-item, caregiver questionnaire that quantifies the severity of the ASD core symptom; and the Autism Parenting Stress Index (APSI; [36]), a13-item parent-rated measure assesses parenting stress in three categories: core social disability, difficult-to-manage behavior, and physical issues.

### 2.3. Definition of ‘Placebo Responders’

A positive response to treatment was defined as (i) a rating of either 1 (very much improved) or 2 (much improved) in the CGI-I; (ii) >25% reduction in the total score on the HSQ-ASD; (iii) >15% reduction in the total score on the SRS-2; (iv) >25% reduction in the total score on the APSI, following treatment. Participants who had a positive response in at least 2 of the 4 outcome measures after being treated with the placebo were defined as placebo responders.

### 2.4. Assessments of Possible Mediators of Placebo Response

To explore possible mediators of the placebo response, we used demographic data of the families, baseline assessments scores, and a specifically designed retrospective questionnaire (the Experience and Expectations Checklist) that taps possible mediators of the placebo by proxy response. Participant characteristics appear in Table 1.

### 2.5. Experience and Expectations Checklist

This 29-item questionnaire (Table 2) was designed and validated for this study. It assesses possible mediators of the placebo by proxy response in 8 domains (Table 3): (1) parental expectations; (2) child’s comprehension of the treatment purpose; (3) previous positive experiences with similar treatment as an indication for behavioral conditioning; (4) parental locus of control; (5) quality of the patient–physician relationships; (6) relative severity of symptoms at baseline, to assess likelihood for regression to the mean; (7) fluctuations in symptom severity at baseline; and (8) adherence to study medications. The questionnaire was administered in Hebrew to participants of the CBA trial who received the placebo in one of the two treatment periods and had at least two valid outcome measures (*n* = 88). The questionnaire was emailed to the participants after the termination of the original study. The internal consistency of the multiple item domains—parental expectations, child’s comprehension of the treatment purpose, and parental locus of control—was found to be ≥ 0.6 (Cronbach’s alpha, acceptable, Table 3).

### 2.6. Statistical Analyses

The internal consistency of the Experience and Expectations Checklist subscales was examined by Cronbach’s alpha. The Mann–Whitney non-parametric test was used to examine the effect of the Experience and Expectations Checklist subscale scores, which are rank variables of the placebo response. Comparing continuous variables between the placebo responders and non-responders was assessed using the two-sample *t*-test. Testing the association between the categorical variables and the placebo response was examined using either the Pearson Chi-square test or the Fisher’s exact test. To assess the effect of several variables on placebo response, a logistic regression model was applied. Analyses were performed using IBM SPSS^®^ version 25, 2017 (IBM Corp., Armonk, NY, USA). All P values were two-sided. A *p*-value ≤ 0.05 was considered statistically significant.

## 3. Results

### 3.1. Placebo Response in the CBA Trial

Overall, 19 participants (22%) had a positive response (as defined in the methods) in at least 2 of the 4 outcome measures following treatment with the placebo (Table 4).

The placebo response was higher in measurements of behavior [HSQ-ASD (35%), and CGI-I (22%)] and the related parental stress assessment (APSI, 22%) compared with the SRS-2, an assessment of the severity of the core symptoms (14%). In the three parental questionnaires (HSQ-ASD, SRS-2, and APSI), the placebo response was higher in the first treatment period compared with the second treatment period. This difference between treatment periods was not demonstrated in the physician assessment (CGI-I).

### 3.2. Associations between Placebo Response and Baseline Characteristics

There was no association between placebo response and the participants’ age, sex, and level of function at baseline, including the VABS composite score, the ADOS comparison score, the CARS total score, and the SCQ total score (Table S1).

### 3.3. Associations between Placebo Response and Symptom Severity at Baseline

There was no significant association between the placebo response and the severity of the participants’ symptoms at baseline, as assessed by the CGI-S and HSQ-ASD (for behavior), SRS-2 (for core symptoms), and APSI (for parental stress related to the child’s condition) (Table S2).

### 3.4. Associations between Placebo Response and Participant’s Experience and Expectations

**Figure 2:**
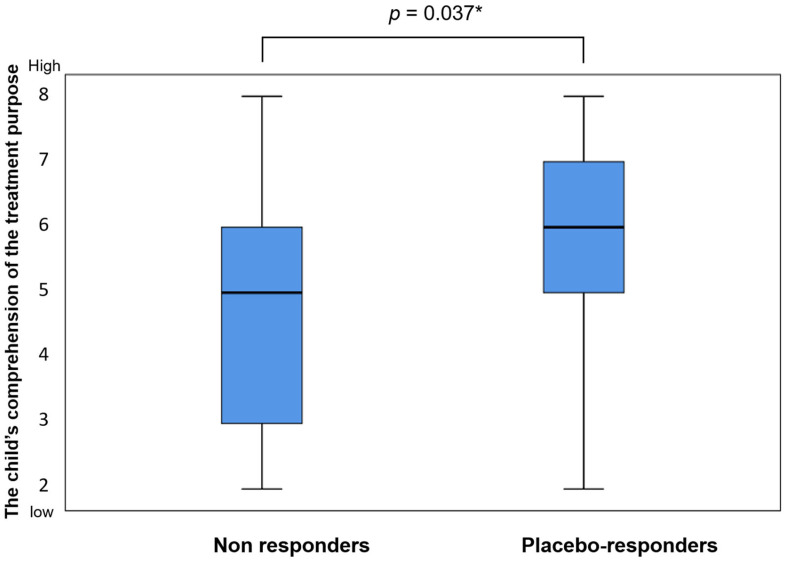
The child’s comprehension of the treatment purpose in placebo responders and non-responders and the distribution of the scores in the domain of the child’s comprehension of the treatment purpose in placebo-responders and non-responders. The box plot represents the median (inside the box) and the interquartile range (25th to 75th percentile). The bars span between the minimal and maximal values. * Mann–Whitney test.

The parents of all 88 children who received the placebo in the CBA trial and had valid outcomes in at least 2 outcome measures were asked to complete the Experience and Expectations Checklist. The parents of 67 children completed the questionnaire (76%). Of the 67 participants, 13 (19%) had a positive response to the placebo for at least 2 outcome measures. Table 5 presents the association between the placebo response and each of the 8 domains tapped by the Experience and Expectations Checklist. The child’s comprehension of the treatment purpose was the only domain that was significantly associated with the placebo response (*p* = 0.037, Figure 2).

Notably, the child’s comprehension of the treatment purpose was positively associated with the placebo response, even after controlling for age and adaptive level (VABS composite score); *p* = 0.013, adjusted odds ratio = 1.9 per 1 point in the domain score (scores range between 2 and 8), and 95% confidence interval =1.15–3.16.

Among 18 children who had no ability to understand the treatment purpose, according to their parents (domain score = 2–3), only 1 (6%) responded to the placebo, as opposed to 12 (25%) of the 49 who could understand the treatment purpose, at least partially (domain score = 4–8).

A relative aggravation of symptoms before treatment onset also increased the likelihood of the placebo response (*p* = 0.053), possibly reflecting an expected regression of the symptoms to the mean. No other domains, including parental expectations, were associated with the placebo response (Table 5).

## 4. Discussion

The current retrospective study assessed possible mediators of the placebo response in participants in the CBA trial, a placebo-controlled trial of the use of cannabinoids to alleviate behavioral problems in children with ASD [28]. Approximately half of the participants could reasonably understand the purpose of the study treatment. In all of the participants, the outcome assessments were based on parental reports, reflecting a placebo by proxy response [19,37]. We found that the main contributor to the placebo response was the child’s ability to understand the treatment purpose, as perceived by the parents retrospectively. The child’s comprehension of the treatment purpose was associated with the placebo response, even after controlling for age and adaptive level. The likelihood of the placebo response was doubled (adjusted OR = 1.9) for each additional point in the child’s comprehension domain score (scores range between 2 and 8).

Our findings are in line with a meta-analysis of 22 studies in children and adults with intellectual disability, which found a higher placebo by proxy response in individuals with a higher IQ [38].

The only other factor that tended to be associated with placebo response in our study was a relative aggravation of symptoms before the study onset, as compared with the average condition of the child in the previous two years. This finding likely reflects an expected course of regression of symptoms to the mean [8].

The placebo by proxy response is estimated to be higher when assessing outcomes that are more subjective, such as disruptive behavior and social deficits, as evaluated in the CBA study. These outcomes are substantially impacted by the interactions with parents and other proxies. Hence, parents’ expectations from the treatment might alter their behavior toward their child, which in turn, will lead to a change in the child’s behavior as a “self-fulfilling prophecy” [39,40].

Notably, as opposed to our hypothesis and suggestions in previous reports [41,42,43], we did not find any association between parental expectations and placebo response. This finding is particularly interesting in the context of cannabinoid treatment. Many studies have demonstrated a relatively high placebo effect to cannabis treatment [23,24,25,26,27], which is often attributed to its perceived high efficacy in the public opinion, supported by reports in the mass media and lay press. Nevertheless, we found no association between placebo response and parental opinion regarding medical cannabis or their previous exposure to children with autism who were successfully treated with medical cannabis. Additionally, we found no associations between placebo response, parental locus of control, adherence to study medications, and the quality of the parent–physician relationships. However, there may be additional parental factors that contribute to the placebo by proxy response that were not assessed in this study.

*Subject characteristics* that have been previously demonstrated to contribute to greater placebo response in adult patients with chronic pain include higher subject expectations [17,18], high adherence to the study medication [17], and larger fluctuations in the symptoms at baseline [19], with no impact of the participants’ sex on the magnitude of the response [20].

*Study characteristics* that have been associated with higher placebo response include longer follow-up [44], larger sample size [45], and a parallel-group design, as opposed to crossover studies [45]. A recent meta-regression analysis [21] of 24 placebo-controlled trials (2229 patients in the placebo arms) of antidepressants for major depressive disorder in children and adolescents demonstrated a higher placebo response rate in clinician ratings (45%) compared with self-rating scales (26%). A higher number of study sites was associated with a slightly higher placebo response rate. The study duration was not significantly associated with the placebo response rate. In a meta-analysis of placebo efficacy in childhood and adolescence migraines [22], factors associated with a lower placebo response rate included crossover trials (vs. parallel-group trials), single-center (vs. multicenter) trials, and small sample size. Age and sex were not associated with the placebo response.

In concordance with a previous study which assessed the response to risperidone, we did not find associations between the placebo response and the severity of the ASD core symptoms or the child’s adaptive level [14,46]. However, a study which assessed the response to citalopram demonstrated a higher placebo response in children with milder baseline symptom severity [47], while studies which assessed the response to balovaptan demonstrated a higher placebo response with greater baseline symptom severity [14,48]. Other domains that were suggested previously to be associated with placebo response and were not found to be associated with the placebo by proxy response in our study include younger age [38], high adherence to the study medication [17], larger fluctuations in the symptoms at baseline [19], and the trust and positive relationship with the clinician [49].

Interestingly, while the placebo effect has been demonstrated to be higher following an active treatment compared to when given for the first time [50,51], we found that in our cross-over trial, the placebo response was higher when given in the first time period (Table 4). Possible explanations include a relatively lower impact of behavioral conditioning in the placebo by proxy response and a higher placebo effect in the first compared with the second period in cross-over trials, resulting from the fear of crossing over to a less effective treatment, after an improvement in the first treatment period.

Finally, this study has several limitations. It is a retrospective study, and the parents’ response to the Experience and Expectations Checklist may not reflect their real-time expectancies at the time of the study onset. The assessment of the child’s comprehension of the treatment purpose was based on the parent report only and was not assessed directly. The internal consistency of the checklist was not robust for some of the subscales and the number of participants was not large enough to evaluate the impact of other possible mediators of the placebo by proxy response.

Future studies should prospectively assess the parent’s and child’s expectations and the child’s comprehension of the treatment purpose, using a structured interview with both the parent and the child. The investigators should standardize the language they use to explain the possible benefits and risks of the treatment, directly assess the expectancies of the child and the parent before treatment onset, and later assess whether they believe the participant received a placebo or an active treatment.

## 5. Conclusions

Despite the importance of the placebo response in clinical and research settings, studies investigating the mediators of the placebo response in children with disabilities are sparse. In adults with normal cognitive functioning, treatment expectation is one of the main mediators of the placebo response. However, in children with disabilities, the association between the child’s ability to understand the purpose of the treatment and the placebo response has not been previously explored. In a cohort of children and adolescents with ASD, we found that the placebo response was mainly associated with the child’s comprehension of the purpose of the treatment, rather than the parent’s expectations. These findings emphasize the importance of explaining the treatment purpose to children with disabilities in a manner that they can understand, rather than talking to caregivers “over their heads”. This strategy may improve the treatment outcome in clinical practice and decrease the differences in the placebo response between participants which potentially lead to study failure in clinical trials.

## Figures and Tables

**Table 1:** Participant characteristics.

	All (*n* = 88)	Group A (*n* = 46)	Group B (*n* = 42)
Treatment—1st period		Placebo	Cannabinoids
Treatment—2nd period		Cannabinoids	Placebo
Sex			
Males *n* (%)	72 (82%)	38 (83%)	34 (81%)
Age			
Mean ± SD	11.7 ± 4.0	11.9 ± 3.8	11.5 ± 4.2
(median, range)	(10.5, 5.1–20.3)	(10.7, 5.8–20.0)	(10.1, 5.1–20.3)
ADOS comparison score			
Mean ± SD	8.8 ± 1.5	8.5 ± 1.7	9.2 ± 1.3
(median, range)	(10.0, 4.0–10.0)	(9.0, 4.0–10.0)	(10.0, 6.0–10.0)
VABS composite score			
Mean ± SD	52.4 ± 15.1	52.7 ± 15.4	52.2 ± 15.0
(median, range)	(51.0, 26.0–102.0)	(51.0, 26.0–102.0)	(54.5, 28.0–89.0)
CARS total score			
Mean ± SD	45.5 ± 8.4	45.4 ± 8.5	45.6 ± 8.4
(median, range)	(47.8, 30.5–59.0)	(47.8, 30.5–59.0)	(48.5, 31–57.5)
SRS T score			
Mean ± SD	83.7 ± 8.6	84.7 ± 7.9	82.7 ± 9.4
(median, range)	(87, 58–91)	(87, 58–91)	(87, 62–91)
Total SCQ score			
Mean ± SD	19.3 ± 6.4	19.3 ± 5.8	19.4 ± 7.0
(median, range)	(20, 7–33)	(20, 8–28)	(19.5, 7–33)
	**All (*n* = 67)**	**Group A (*n* = 32)**	**Group B (*n* = 35)**
Treatment—1st period		Placebo	Cannabinoids
Treatment—2nd period		Cannabinoids	Placebo
Parental expectations ^a^			
Mean ± SD	39.3 ± 4.7	39.1 ± 4.6	39.5 ± 4.9
(median, range)	(39, 27–49)	(38.5, 27–47)	(39, 30–49)
Child’s comprehension of the treatment purpose ^b^			
Mean ± SD	4.9 ± 2.0	5.2 ± 1.8	4.7 ± 2.1
(median, range)	(5, 2–8)	(5, 2–8)	(5, 2–8)
Experience with similar treatment ^c^			
Mean ± SD	6.7 ± 1.5	7.0 ± 1.5	6.4 ± 1.4
(median, range)	(7, 4–11)	(7, 4–11)	(6, 4–9)
Parental locus of control ^d^			
Mean ± SD	17.5 ± 2.2	17.7 ± 2.2	17.4 ± 2.2
(median, range)	(17, 13–24)	(18, 14–24)	(17, 13–22)
Patient-physician relationships ^e^			
Mean ± SD	2.7 ± 0.7	2.8 ± 0.7	2.7 ± 0.8
(median, range)	(3, 1–4)	(3, 1–4)	(3, 1–4)
Relative severity at baseline ^e^			
Mean ± SD	2.7 ± 1.1	2.8 ± 1.1	2.5 ± 1.0
(median, range)	(3, 1–4)	(3, 1–4)	(3, 1–4)
Fluctuations in severity at baseline ^e^			
Mean ± SD	2.8 ± 0.8	2.8 ± 0.8	2.7 ± 0.8
(median, range)	(3, 1–4)	(3, 1–4)	(3, 1–4)
Adherence to study medications ^f^			
Mean ± SD	3.8 ± 0.5	3.8 ± 0.4	3.7 ± 0.5
(median, range)	(4, 2–4)	(4, 3–4)	(4, 2–4)

Baseline characteristics of participants stratified to treatment arms. ADOS-2: Autism Diagnostic Observation Schedule, comparison score of 8–10 indicated severe autistic symptoms; CARS: Childhood Autism Rating Scale, scores above 36.5 are indicative of severe ASD; SCQ: Social Communication Questionnaire score ≥15 indicated possible ASD; SRS: Social Responsiveness Scale, total score ≥75 indicated severe autistic symptoms; VABS: Vineland Adaptive Behavior Scale, composite score ≤70 indicated low adaptive level. ^a^ Scores range between 15 (lowest) to 60 (highest) expectations. ^b^ Scores range between 2 (no comprehension) to 8 (full comprehension). ^c^ Scores range between 3 (minimal) to 12 (substantial) positive previous experience with similar treatment. ^d^ Scores range between 6 (external) to 24 (internal) locus of control. ^e^ Higher scores indicate better patient–physician relationships, higher relative severity at baseline, and larger fluctuations in severity at baseline. ^f^ Scores range between 1 (poor) to 4 (excellent) adherence.

**Table 2:** Experience and Expectations Checklist.

#	Statements	Strongly Agree (4)	Agree (3)	Somewhat Agree (2)	Disagree (1)
1	I can influence the health of my child.				
2	When my child is not feeling well, medication usually helps.				
3	When I am not feeling well, medication usually helps me.				
4	I tend to look at the glass as “half-full”.				
5	When I give my child medicine (e.g., to relieve pain) he/she usually understands that the treatment may help him/her.				
6	My child can understand that the treatment given in the study could improve his/her communication difficulties and regulate his/her behavior.				
7	I have full confidence in the physicians treating my child.				
8	When we started the study treatment, my child’s condition was worse compared to his/her average condition in the previous two years.				
9	When I have a problem, I usually manage to solve it.				
10	I can help my child **greatly** improve his or her communication difficulties and repetitive behavior.				
11	I can help my child **greatly** improve his or her behavioral regulation.				
12	During the months that preceded the study onset, my child’s condition varied greatly (very good weeks and very difficult weeks)				
13	When we joined the study, I believed that the treatment given in the study may **greatly** improve the communication difficulties and repetitive behavior of my child.				
14	When we joined the study, I believed that the treatment given in the study may **greatly** improve the regulation of my child’s behavior.				
15	During the study we were very careful to give the oil drops at the right time and in the right amount.				
16	Before we joined the study, I was exposed to publications that described children who were helped by medical cannabis treatment.				
17	Before we joined the study, I personally knew a child with autism who was helped by medical cannabis treatment.				
18	I do not despair easily.				
19	Before we joined the study, one of us (parents) feared that the treatment given in the study would not help.				
20	When I start a new treatment, I am usually afraid of possible side effects.				
21	If you put in enough effort, you can achieve almost any goal.				
22	Medication that my child received in the past due to behavioral difficulties caused **severe** side effects and was discontinued.				
23	During the year prior to the study onset, my child received a medication that helped him/her with his/her behavioral difficulties.				
24	‘Natural’ treatments (diets, supplements or herbal or animal-based treatments) are usually more effective than ‘chemical’ treatments (medications).				
25	‘Natural’ treatments are usually safer than ‘chemical’ treatments.				
26	Medical cannabis is usually more effective than ‘chemical’ drugs.				
27	Medical cannabis is usually safer than ‘chemical’ drugs.				
28	In the two years preceding the study, my child has been treated with at least two ‘natural’ treatments.				
29	In the two years preceding the study, at least one natural treatment has been very helpful to my child.				

The questionnaire presents various statements. Please indicate to what extent you agree with each statement.

**Table 3:** Internal consistency of the 8 domains tapped in the Experience and Expectations Checklist.

Domain	Related Items	Internal Consistency Cronbach’s Alpha
Parental expectations	15 items: 3,4,13,14,16,17,19, 20, 22, 24–29	0.6
Child’s comprehension of treatment purpose	#5, #6	0.86
Previous positive experience with treatments	#2, #23, #29	0.19
Parental locus of control	6 items: 1,9,10,11,18,21	0.65
Quality of the patient–physician relationships	#7	N/A
Relative aggravation of symptoms at baseline	#8	N/A
Fluctuations in symptom severity at baseline	#12	N/A
Adherence to study medications	#15	N/A

N/A, not applicable.

**Table 4:** Positive responses to placebo [*n* (%)] in each assessment and treatment period.

	Period 1 (12 Weeks)	Period 2 (12 Weeks)	Period 1 + Period 2
Assigned to receive placebo (*n*)	50	50	100
Completed the treatment period (*n*)	47	44	91
CGI-I, valid rate (*n*)	47	44	91
CGI-I, positive response [*n* (%)]	10 (21%)	10 (23%)	20 (22%)
HSQ-ASD, valid rate (*n*)	39	35	74
HSQ-ASD, positive response [*n* (%)]	18 (46%)	8 (23%)	26 (35%)
SRS-2, valid rate (*n*)	36	30	66
SRS-2, positive response [*n* (%)]	7 (19%)	2 (7%)	9 (14%)
APSI, valid rate (*n*)	42	37	79
APSI, positive response [*n* (%)]	11 (26%)	6 (16%)	17 (22%)
At least one valid outcome measure (*n*)	47	44	91
A positive response in at least 1 outcome measure [*n* (%)]	28 (60%)	19 (43%)	47 (52%)
At least 2 valid outcome measures (*n*)	46	42	88
A positive response in at least 2 outcome measures [*n* (%)]	13 (28%)	6 (14%)	19 (22%) **placebo responders**

**Table 5:** Association between participant characteristics and placebo response.

Domain	Z Score	*p* Value *
Parental expectations	−0.628	0.530
Child’s comprehension of treatment purpose	−2.086	**0.037**
Previous positive experience with treatments	−0.665	0.506
Parental locus of control	−0.504	0.614
Quality of the patient–physician relationships	−0.052	0.958
Relative aggravation of symptoms at baseline	−1.933	**0.053**
Fluctuations in symptom severity at baseline	−0.103	0.918
Adherence to study medications	−0.756	0.450

* The effect of the *Experience and Expectations Checklist* subscales scores on the placebo response was assessed using the Mann–Whitney test. Bold font indicates significant values.

## Data Availability

The authors declare that the data supporting the findings of this study are available within the paper and its Supplementary Materials. The remainder of data is available from the corresponding author upon reasonable request.

## Supplementary Materials

The following supporting information can be downloaded at: https://www.mdpi.com/article/10.3390/jcm12093098/s1, Table S1: Associations between placebo response and baseline characteristics; Table S2: Associations between placebo response and symptom severity at baseline; Table S3. STROBE Statement—checklist of items that should be included in reports of observational studies.

## Appendix A. Placebo Response in Previous Studies with Medical Cannabis

A meta-analysis of 47 controlled studies of cannabinoid treatment in chronic noncancer pain demonstrated a very small differences between cannabinoids and placebo (*n* = 9958 participants) [23]. Two more recent meta-analyses (N = 5174), [24] and (N = 1534) [25] demonstrated a comparable minimal benefit over placebo. Similarly, a minimal advantage of cannabinoids over placebo was found in a recent meta-analysis of chemotherapy-induced nausea and vomiting [26].

Another meta-analysis of 23 randomized controlled trials (2551 participants) in depression, 17 randomized controlled trials (605 participants) in anxiety, and 6 randomized controlled trials (281 participants) in psychosis concluded that cannabis preparations had no benefit over placebo [27].
